# Circulating biomarkers of extracellular matrix dysregulation are associated with adverse post-stage 2 outcomes in infants with single ventricle heart disease

**DOI:** 10.1038/s41598-023-43562-4

**Published:** 2023-09-28

**Authors:** Benjamin S. Frank, Debmalya Nandy, Ludmila Khailova, Max B. Mitchell, Gareth J. Morgan, Mark Twite, Michael V. DiMaria, Jesse A. Davidson

**Affiliations:** 1 Section of Cardiology, University of Colorado Department of Pediatrics, 13123 E. 16th Ave, Box B100, Aurora, CO 80045 USA; 2 Center for Innovative Design and Analysis, University of Colorado Department of Biostatistics and Informatics, Denver, CO USA; 3 University of Colorado Department of Surgery, Denver, CO USA; 4 University of Colorado Department of Anesthesiology, Denver, CO USA

**Keywords:** Biomarkers, Paediatric research, Translational research

## Abstract

Children with single ventricle heart disease (SVHD) experience morbidity due to inadequate pulmonary blood flow. Using proteomic screening, our group previously identified members of the matrix metalloproteinase (MMP), tissue inhibitor of metalloproteinase (TIMP), and fibroblast growth factor (FGF) families as potentially dysregulated in SVHD. No prior study has taken a targeted approach to mapping circulating levels of these protein families or their relationship to pulmonary vascular outcomes in SVHD. We performed a prospective cohort study of 70 SVHD infants pre-Stage 2 palliation and 24 healthy controls. We report targeted serum quantification of 39 proteins in the MMP, TIMP, and FGF families using the SomaScan platform. Clinical variables were extracted from the medical record. Twenty of 39 tested proteins (7/14 MMPs, 2/4 TIMPs, and 11/21 FGFs) differed between cases and controls. On single variable testing, 6 proteins and no clinical covariates were associated with both post-Stage 2 hypoxemia and length of stay. Multiple-protein modeling identified increased circulating MMP 7 and MMP 17, and decreased circulating MMP 8 and FGFR2 as most associated with post-Stage 2 hypoxemia; increased MMP 7 and TIMP 4 and decreased circulating MMP 1 and MMP 8 were most associated with post-operation length of stay. The MMP, TIMP, and FGF families are altered in SVHD. Pre-Stage 2 imbalance of extracellular matrix (ECM) proteins—increased MMP 7 and decreased MMP 8—was associated with multiple adverse post-operation outcomes. Maintenance of the ECM may be an important pathophysiologic driver of Stage 2 readiness in SVHD.

## Introduction

Single ventricle heart disease (SVHD) is a severe form of congenital heart disease (CHD) affecting between 2 and 8 in 10,000 live births in the United States in which patients are born with a single functional pumping chamber^1,2^. While there is no cure for SVHD, with staged surgical palliation many affected children can survive into adulthood. Beginning in the first week after birth (following a Stage 1 palliation for many patients) and continuing to the Stage 2 operation, the interstage period is a crucial time for children with SVHD. During interstage, children have an unobstructed pathway from the single ventricle to the systemic circulation and a pressure-limiting source of flow to the pulmonary circulation—most often a shunt between the aorta or right ventricle and the pulmonary arteries. The Stage 2 operation that ends the interstage period—achieved via a Glenn or hemi-Fontan anastomosis—is typically performed at 4–6 months of age^1^. In this operation, the patient’s mechanism of blood flow to the lungs is surgically converted from an active process driven by ventricular systole to a passive process via a direct connection of the superior vena cava to the pulmonary arteries. This abrupt change makes the peri-Stage 2 period the optimal time to evaluate both pre-operative markers of interstage pulmonary vascular growth and the post-operative sufficiency of the pulmonary vasculature to accept passive blood flow.

Among children who undergo Stage 2 palliation, up to 25% experience a complication in the immediate post-operative period that is directly linked to pulmonary vascular disease causing impaired pulmonary blood flow. These include severe hypoxemia, respiratory failure, and persistent pleural effusions^3,4^. Despite this high clinical burden, there are no validated metrics or biomarkers available to reliably identify SVHD patients at particular risk for pulmonary vascular inadequacy. Diagnosis and treatment of pathologic pulmonary vascular development are therefore limited due to poor understanding of the underlying mechanisms.

Using targeted cardiovascular proteomic profiling of infants undergoing Stage 2 palliation, our group has previously demonstrated a distinct serum proteomic phenotype among SVHD infants who manifest pulmonary vascular inadequacy compared to those with better pulmonary blood flow^5^. Through a limited evaluation of circulating levels of a multitude of protein families, we identified members of the matrix metalloproteinase (MMP), tissue inhibitor of metalloproteinase (TIMP), and fibroblast growth factor (FGF) families as potentially important in the distinct proteomic fingerprints identified. The MMPs and their inhibitors, the TIMPs, are primary endogenous modulators of extracellular matrix production and activity of several members has been linked to cardiac and pulmonary pathology in a variety of populations including adults with a Fontan circulation^6–10^. The FGFs are a heterogenous family of growth factors that includes several members with activity tied to angiogenesis and fibrosis^11^. No prior study has taken a comprehensive approach to mapping the circulating levels of these protein families in the pediatric SVHD population, particularly those still undergoing staged palliation. Here we present a prospective, cohort study of targeted mapping of the circulating MMP, TIMP, and FGF protein families in SVHD infants undergoing Stage 2 palliation. We hypothesized that infants with SVHD would have different circulating levels of multiple measured proteins compared to healthy controls and that a panel containing a subset of the MMP, TIMP, and FGF proteins would be able to predict patients at greater risk for post-Stage 2 complications associated with insufficient pulmonary blood flow.

## Methods

### Subjects

The Colorado Multiple Institution Review Board approved this study and all research was performed in accordance with the Declaration of Helsinki. Written informed consent was obtained from the study subjects’ parents in all cases. In this prospective cohort study, we approached all subjects age 31 days to 2 years at Children’s Hospital Colorado in Denver, Colorado, USA from 2018 to 2021 with SVHD either undergoing pre-Stage 2 catheterization or Stage 2 palliation without plans for cardiac catheterization for enrollment^12^. When possible, subjects were approached prior to their pre-Stage 2 catheterization. For those not approached prior to catheterization this occurred either due to catheterization occurring prior to the research team having an opportunity to obtain informed consent or because a pre-Stage 2 catheterization was not deemed clinically necessary. We considered any form of superior cavo-pulmonary anastomosis (Glenn or Hemi-Fontan operation) as a Stage 2 independent of whether a patient had previously undergone a Stage 1 procedure. We excluded patients with a so-called 1.5 ventricle repair (those with a persistent, additional pulsatile source of pulmonary blood flow).

Control subjects were identified from the main operating room schedule at Children’s Hospital Colorado. Control subject inclusion criteria were patients greater than 4 kg, 3–12 months of age, undergoing anesthesia for an elective, non-cardiac procedure with a plan for a clinically indicated peripheral IV. We excluded potential control subjects if they had any known or suspected cardiac, pulmonary, infectious, or genetic abnormalities.

### Clinical data

The reported clinical data were extracted from the electronic medical record including cardiac diagnosis and dominant ventricle morphology, growth index (weight in kilograms divided by height in meters)^13^, medication regimen, catheterization results, and hospitalization duration (Epic Systems, Verona, WI). Post-operative oxygen saturations were extracted at 1-min intervals from the BedMaster hemodynamic monitoring system in the cardiac intensive care unit (Anandic Medical Systems, Feuerthalen, Switzerland). Study data were collected and managed using REDCap electronic data capture tools hosted at University of Colorado. We identified a priori clinical variables to evaluate the relationship between protein biomarkers of interest and subjects with more or less favorable clinical outcomes. The primary variables of interest were percent of time in the first 48 post-operative hours with clinically significant hypoxemia, defined as an oxygen saturation below 70% (48 h Low Sat%), and post-operative length of stay (LOS). We attempted to mitigate the effect of “noise” in the oxygen saturation data by analyzing % time below a clinically relevant threshold, rather than simply calculating mean saturation, since mean values are more affected by outlier measurements. Secondary variables included pre-Stage 2 pulmonary vascular resistance and post-Stage 2 endotracheal intubation time (ETT), chest tube days, discharge home on a phosphodiesterase type 5 inhibitor (PDE-5i) and volume of chest tube drainage.

### Sample collection and protein analysis

All pre-operation whole blood samples were obtained under general anesthesia. Samples were collected in the early morning after a period of fasting for most patients. For SVHD subjects enrolled at a pre-Stage 2 catheterization, systemic vein samples were collected prior to any procedural interventions. For subjects enrolled at other times, a systemic venous sample was obtained on the day of Stage 2 palliation before first surgical incision. Control subject samples were obtained from a systemic vein (peripheral intravenous line) after induction of anesthesia at the time of IV placement. All samples were processed for serum at the time of collection, aliquoted, and stored at −80 °C until batch analysis.

Samples underwent targeted proteomic phenotyping using an aptamer-based assay (SomaLogic, Boulder, CO) performed by SomaLogic at their laboratory in Colorado, USA. The detailed methods of this assay and prior validation data have been previously published^14^. Briefly, each protein target has its own binding reagent comprised of fluorescent tag-labeled modified DNA, referred to as a modified aptamer. Each serum sample was incubated with a mixture of the modified aptamers generating aptamer-protein complexes. After elimination of unbound aptamers, the bound aptamers were then quantified using a hybridization array. Quality control confirmed intra-assay and inter-assay variability of < 5%. Raw abundance measurements were log-transformed, centered to mean 0 and scaled to variance 1. Higher expression values correspond to higher protein levels but are not an absolute quantification of protein concentrations. The SomaLogic assay can measure the relative abundance of up to 7000 proteins per sample. Prior to assay, we a priori identified 39 candidate proteins of interest for targeted analysis based on membership in the MMP, TIMP, and FGF families, or status as FGF-interacting: MMP 1–3, 7–10, 12–14, 16–17, 19–20, TIMP 1–4, FGF 1–10, 12, 16–20, 22–23, FGFR1, FGFR2, and FGF P1. For simplicity, we have referred to both true members of the FGF family and its associated proteins FGFR1, FGFR2, and FGF P1 as “FGFs”.

### Statistical analysis

Demographic and clinical variables were summarized using descriptive statistics as indicated by the distribution of the data. Statistical analyses were performed in R. The Wilcoxon rank sum test was used to compare continuous variables and Fisher’s exact testing was used for categorical variables. Analysis of the distribution of the protein abundance data revealed that whether a patient received heparin prior to sample acquisition affected the level of several proteins of interest. Therefore, we performed the case–control analysis in 2 ways. First, we considered all SVHD cases and healthy controls using multiple linear regression for each protein target (n = 39) to regress out the heparin effect. In this analysis, protein abundance was the outcome of interest and case–control status was a candidate predictor adjusted for sex and heparin status. In a second analysis, we directly compared the protein abundances of the subset of cases who did not receive heparin to those of the controls (none of whom received heparin).

For the post-operative clinical outcome analysis only the SVHD cases were considered. The a priori primary outcomes of interest were 48 h low sat% and post-operative LOS. In this analysis we first accounted for the effect of heparin status on each individual protein abundance for each subject using a one-way ANOVA model fit. The residuals from these 39 model fits were then considered as adjusted protein abundances and used for all downstream analyses. All continuous predictor variables were log-transformed, mean centered, and variance scaled prior to analysis. For each outcome of interest, we then fit protein-by-protein linear regression models (n = 39) considering the adjusted protein abundance adjusted for demographic covariates including age, sex, ventricular morphology, and growth index (weight in kg/height in m). Proteins with a significant association with the outcome of interest (*p* < 0.05 in the models) were considered for multiple-protein analysis. In the multiple-protein analysis we first considered all the demographic variables and only the proteins significant on single protein testing. We then ran the multiple-protein analysis a second time considering those same variables as well as any of diagnostic variables atrioventricular valve regurgitation, ventricular end diastolic pressure, mean pulmonary artery pressure, and indexed pulmonary vascular resistance that were significantly associated with the outcome as covariates. We considered but then excluded ventricular function as a covariate due to an overwhelming preponderance of subjects having normal function (63 v. 7). To identify the optimal model for predicting the outcomes of interest we considered all possible combinations of the variables for each analysis using all possible subsets regression. We considered adjusted R^2^, Mallow’s C_p_, and Bayesian Information Criterion (BIC) as best subset model selection criteria^15–17^. Among the three models (one selected based on each of the 3 model selection criteria) we performed five-fold cross validation repeated 100 times and selected the winner model based on the one with the smallest average root mean-squared error. Alpha value to determine significance was set at 0.05 for all analyses.

## Results

### Study population

**Figure 1:**
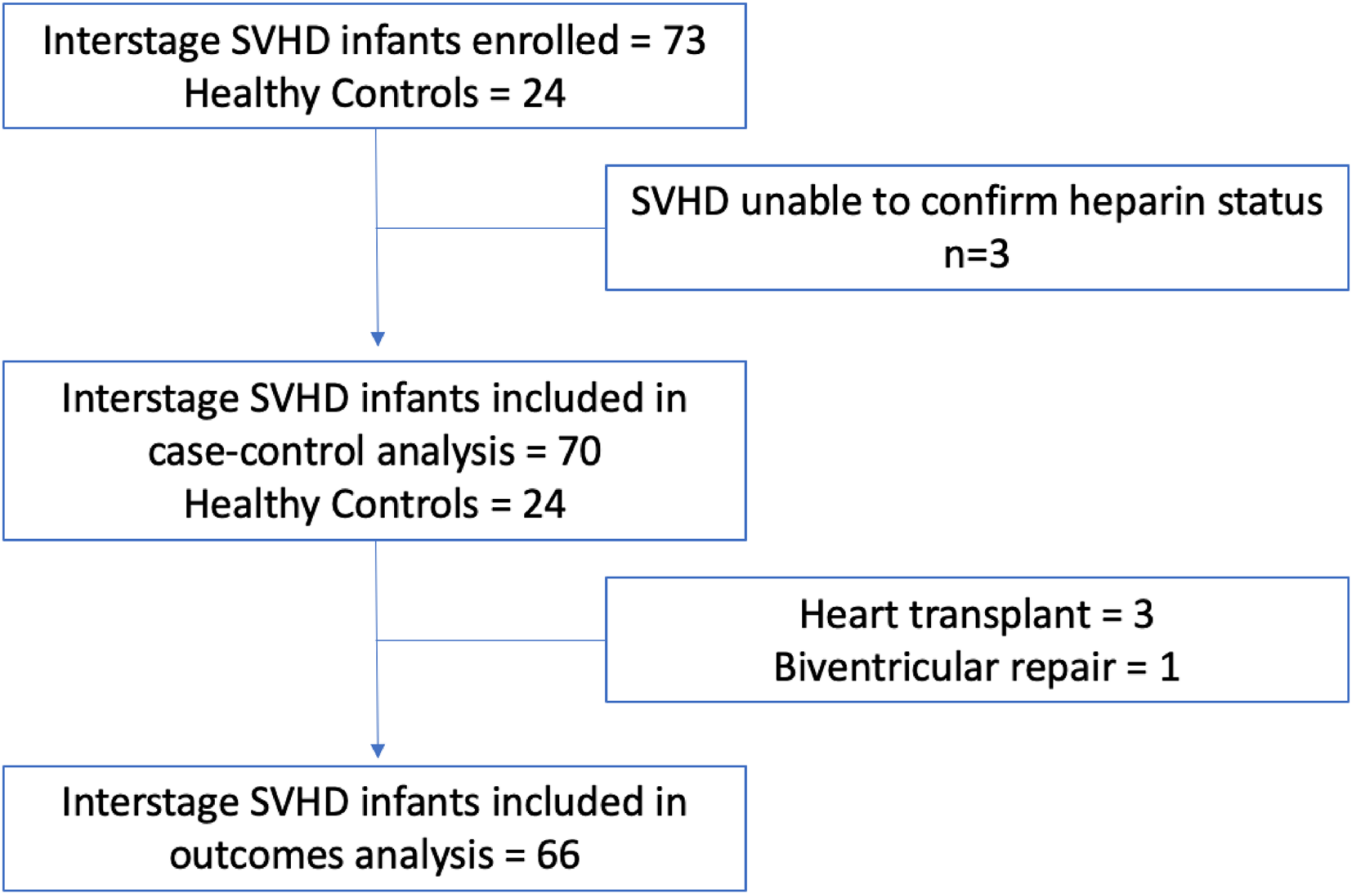
Study diagram. From the original single ventricle cohort of 73, three subjects were excluded from analysis due to inability to confirm heparin status at the time of sampling. Four additional subjects were excluded from the post-operation analysis due to undergoing a non-Stage 2 operation after enrollment.

Seventy-three SVHD cases and twenty-four similar age healthy controls were enrolled (Fig. 1). Pre-operation systemic vein samples were available for all cases and controls. Thirty-seven cases were found to have received a dose of heparin prior to their sample being drawn (each of whom were subjects sampled at pre-Stage 2 catheterization), while 33 cases and all 24 controls had no heparin exposure. Fifteen subjects had their pre-operative sample collected in the operating room. In 3 subjects we were unable to confirm the timing of heparin administration relative to sample collection; those subjects were excluded from further analysis. Therefore, the final pre-operation analysis included n = 70 SVHD cases and n = 24 controls. Four SVHD subjects ultimately did not undergo Stage 2 palliation (3 heart transplantation, 1 biventricular repair), so 66 SVHD subjects were included in the post-operation outcomes analysis. Although abundances were similar between cases with samples collected in the cath lab and OR for most proteins; significantly lower protein levels were seen among the patients with cath collection compared to OR collection for MMP 7, FGF 9, and FGF 20. Control subjects were undergoing surgery with urology (e.g. hypospadias, chordee), orthopedics (e.g. supernumerary digit, hip dysplasia), neurosurgery (e.g. craniosynostosis), ophthalmology (e.g. strabismus), or other surgical teams (e.g. inguinal hernia). Demographics and clinical characteristics are presented in Table 1.

**Table 1:** Demographics and Clinical Characteristics.

	Control (n = 24)	SVHD (n = 70)	*p*-value
Weight (kg)	7.995 (6.580, 10.380)	5.700 (3.800, 10.400)	1.384 E-10
Height (cm)	69.10 (63.50, 80)	61.75 (41, 87)	1.084 E-09
Growth Index (kg/m)	11.69 (10.14, 12.97)	9.38 (7.31, 13.42)	8.60 E-10
Age (days)	248.5 (156, 355)	124 (26, 649)	7.806 E-12
Sex (F)	10 (41.67%)	30 (42.86%)	1
Heparin exposure (Y)	0 (0%)	37 (52.86%)	9.704 E-07
Mean PA pressure (mmHg)		13 (8, 35)	
PVRI (UNITS*M^2^)		2 (0.70, 14)	
Qp (l/min/M^2^)		3.60 (1.40, 11.60)	
Qs (l/min/M^2^)		3.18 (1.92, 8.33)	
Qp/Qs		1.10 (0.30, 3.80)	
System ventricle EDP (mmHg)		6.50 (3, 14)	
PRE-OP PDE-5I (Yes)		4 (5.7%)	
Mean SPO2 for the			
first 48 h (%)		78.60 (67.60, 86.80)	
48H LOW SAT % (%)		4.20 (0.10, 62.60)	
Discharge PDE-5i therapy (Yes)		22 (31.43%)	
Post-op length of stay (days)		7 (4, 182)	
Endotracheal			
Intubation duration (hours)		16 (0, 504)	
Chest tube duration (days)		2 (1, 15)	
Total pleural drainage (ml)		137 (0, 701)	

SVHD = single ventricle heart disease, PA = pulmonary artery, PVRi = indexed pulmonary vascular resistance, Qp = pulmonary blood flow, Qs = systemic blood flow, EDP = end diastolic pressure, SpO2 = oxygen saturation, 48h low sat % = percent time in the first 48 post-operative hours with oxygen saturation < 70%, PDE-5i = phosphodiesterase type 5 inhibitor. Variables expressed as median (range) or n (%).

### Pre-operation differences between cases and controls

**Figure 2:**
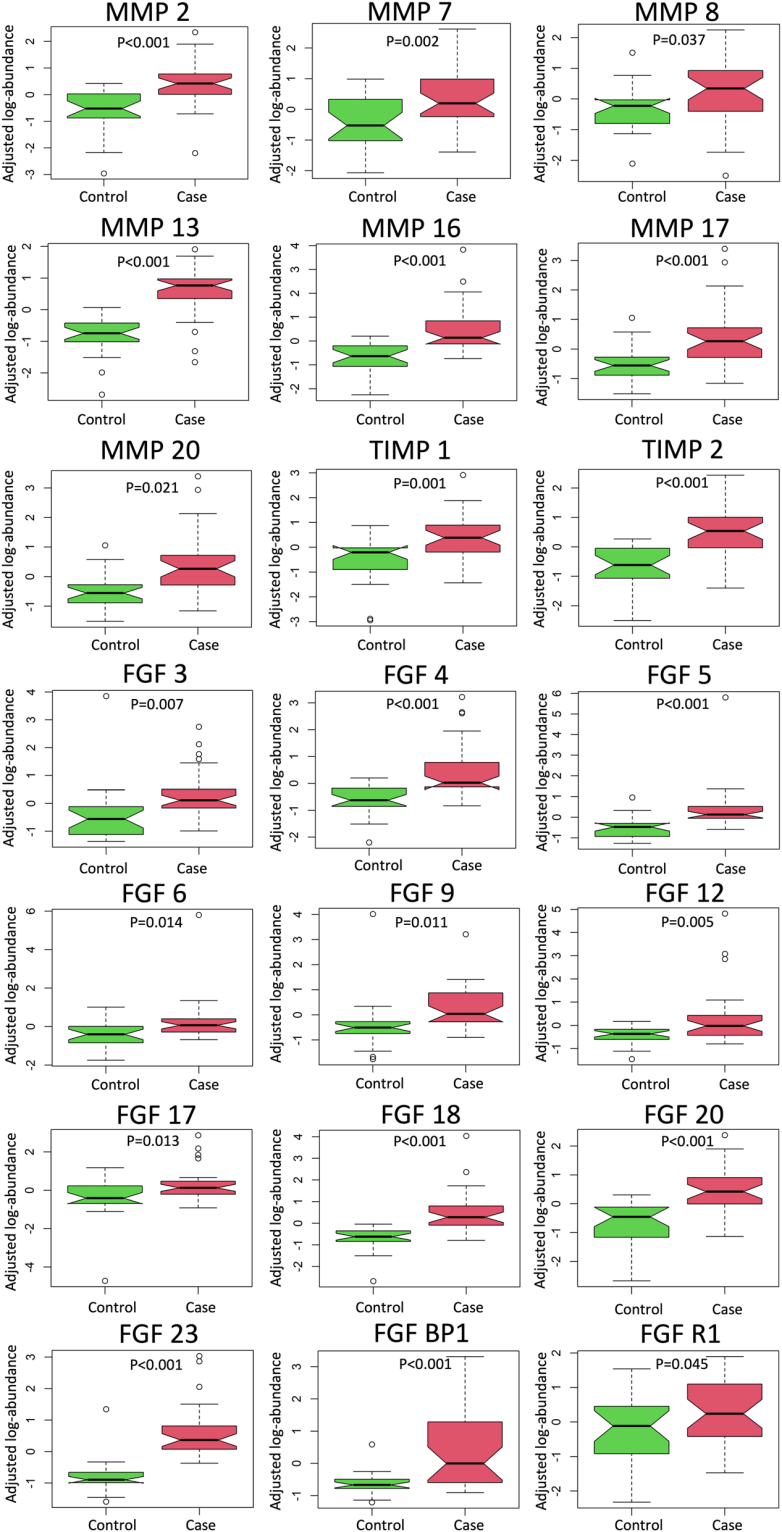
Case control analysis. Box plots depict adjusted log-abundance for proteins that differed between non-heparinized cases and non-heparinized controls. MMP = matrix metalloprotease, FGF = fibroblast growth factor, TIMP = tissue inhibitor of metalloprotease.

We first considered all SVHD cases and evaluated whether circulating levels of any members of the MMP, TIMP, or FGF protein families were associated with case–control status after adjusting for patient sex and heparin status. Case–control status was associated with 20 of the 39 tested proteins, including 7 of 14 MMPs, 2 of 4 TIMPs, and 11 of 21 FGFs (Table 2). Measured abundances were higher in cases than controls for all significant proteins except MMP 20. In the second case–control analysis, considering only the subgroup of cases who did not receive heparin, we identified 21 proteins with different circulating abundances between infants with SVHD and controls (Fig. 2). Notably, the same 20 proteins identified in the heparin-adjusted case–control analysis were again identified in the non-heparin exposed subgroup analysis, with one additional protein noted in the second set (FGF 17).

**Table 2:** Multiple linear regression analysis of SVHD cases versus controls.

Protein	Coefficient	*p*-valuE
FGF 23	1.30E+00	8.60E-09
MMP 13	1.02E+00	8.04E-08
MMP 16	9.41E-01	9.43E-08
FGF 20	8.64E-01	7.65E-07
TIMP 2	1.08E + 00	1.52E-06
FGF 4	7.37E-01	2.37E-05
MMP 2	9.45E-01	3.76E-05
FGFBP1	8.07E-01	3.97E-05
FGF 5	0.810707	0.000147
MMP 17	0.589192	0.001133
MMP 7	0.833692	0.001717
FGF 12	0.714738	0.00247
FGF 18	0.491514	0.00397
FGF 3	0.570472	0.005341
FGF 6	0.628252	0.005475
MMP 20	− 0.48239	0.017481
FGF 9	0.562842	0.018628
TIMP 1	0.58405	0.024967
FGFR1	0.548051	0.03642
MMP 8	0.540144	0.040557

Proteins whose circulating abundance differs between cases and controls after adjusting for sex and heparin status. Positive coefficients indicate higher abundance in SVHD cases than controls whereas negative coefficients indicate higher protein abundance in controls. SVHD = single ventricle heart disease, MMP = matrix metalloprotease, FGF = fibroblast growth factor, TIMP = tissue inhibitor of metalloprotease.

### Post-operative outcomes

The SVHD study population experienced moderate morbidity in the post-Stage 2 period (Table 1). The median patient spent 4.2% of the first 48 post-operative hours with saturation < 70%. The distribution of 48 h low sat% was right skewed such that the mean was 11.9% and the range of observed outcomes varied from 0.1 to 62.6%. Similarly, while the median endotracheal intubation time was 16 h, the range of observed outcomes varied from 0 to 504 h. No patient required an emergent return to the operating room and all patients survived to discharge from our hospital. Fourteen subjects underwent heart catheterization prior to discharge, with persistent hypoxemia the indication in most cases. Twenty-two subjects were discharged home on targeted pulmonary hypertension therapy (PDE-5i in all cases). Median length of stay for the entire SVHD cohort was 7 days (range 4–182 days), and all patients were discharged on supplemental oxygen due to residence at high elevation, per our local protocol. Pre-Stage 2 PVRi demonstrated a positive marginal association with 48 h low sat% (*p* = 0.026) and likelihood of discharge on a PDE-5i (*p* = 0.005) while mean pulmonary artery pressure, ventricular end diastolic pressure, and pre-Stage 2 degree of atrioventricular valve regurgitation were not associated with the primary outcomes.

### Association between protein abundances and post-operation outcomes

**Figure 3:**
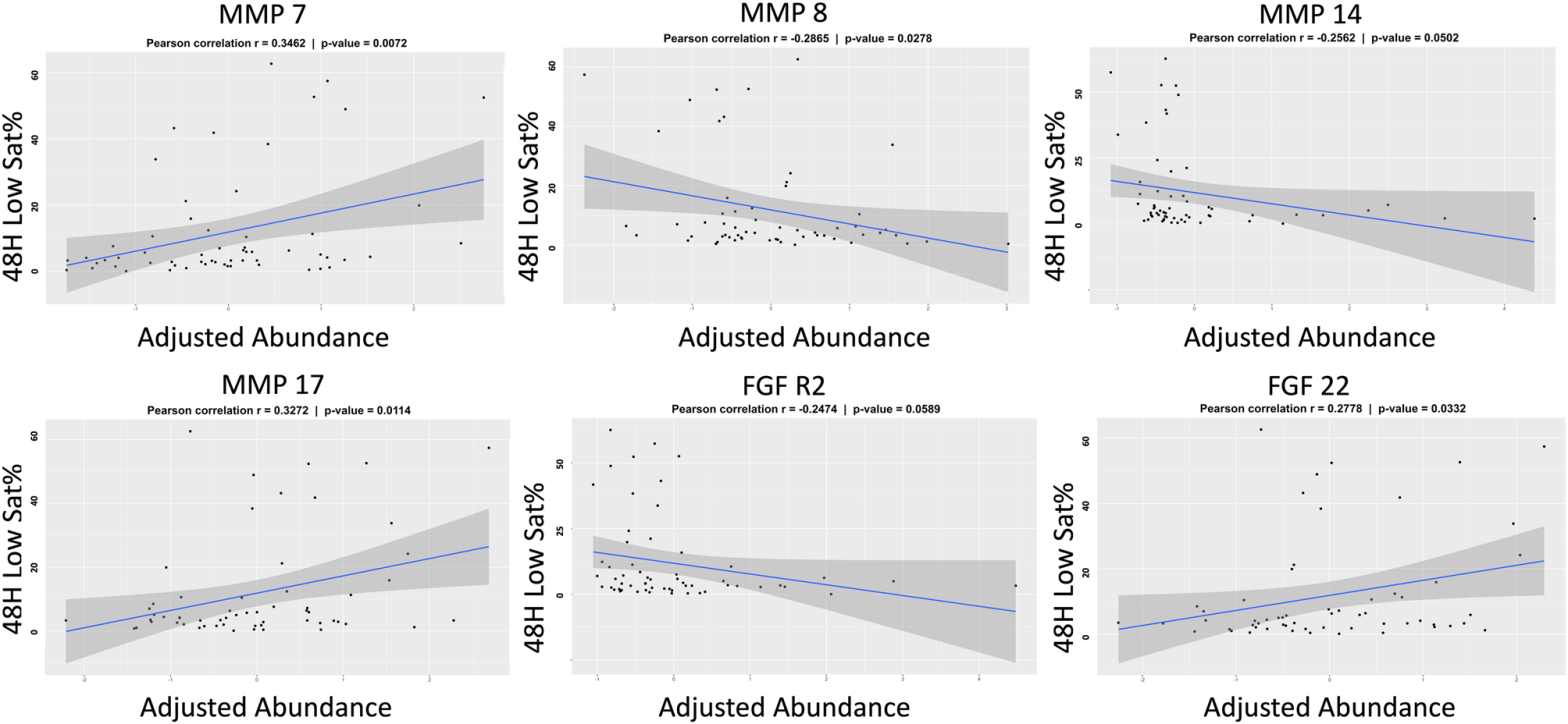
Association between proteins and hypoxemia burden. Scatterplots depict the log transformed, center scaled, heparin-status adjusted protein abundance and post-Stage 2 hypoxemia burden for individual study subjects. Best fit line and 95% confidence interval are overlaid to depict the relationship between the variables. MMP = matrix metalloprotease, FGF = fibroblast growth factor.

We next evaluated whether any of the individual protein abundances were associated with our primary outcome of interest, 48 h low sat%. After controlling for demographic covariates, testing the protein abundances one-by-one there were six that showed a significant linear association with post-operation hypoxemia burden: MMP 7, MMP 8, MMP 14, MMP 17, FGFR2, and FGF 22. Scatterplots depicting the relationship between each adjusted circulating protein abundance and 48 h low sat% for the individual patients are shown in Fig. 3. The six proteins that were significant on protein-by-protein testing along with the clinical variables were then considered for the multiple-protein analysis. The final selected model included four protein abundances (MMP 7, MMP 8, MMP 17, and FGFR2) in addition to growth index and demonstrated a moderate ability to predict the outcome variable (R^2^ = 0.416, adjusted R^2^ = 0.361; Table 3). MMP 7, MMP 17, and growth index levels were higher in subjects with a greater hypoxemia burden while MMP 8 and FGFR2 levels were lower in those who experienced more hypoxemia. Repeating this testing with additional consideration of diagnostic variables atrioventricular valve regurgitation severity, pulmonary vascular resistance, mean pulmonary artery pressure, and ventricular end diastolic pressure as covariates the final winner multiple-protein model demonstrated a positive association between MMP 7, FGF 22, and growth index and 48 h Low Sat% while MMP 8, FGF R2, and age had a negative association with the outcome (full model R^2^ = 0.466, adjusted R^2^ = 0.400).

**Table 3:** Results of multi-protein models evaluating the relationship between serum protein abundances and outcomes of interest.

Protein	Coefficient	*p* value
**48h Low Sat%** adjusted r^2^ = 0.361		
MMP 7	7.07	4.96 E-04
MMP 17	5.53	3.33 E-03
MMP 8	− 3.79	3.72 E-02
FGFR2	− 3.51	5.79 E-02
Growth index	4.64	1.52 E-02
**Length of Stay** adjusted r^2^ = 0.363		
MMP 7	7.63	2.16 E-02
MMP 8	− 7.3	2.71 E-02
MMP 1	− 12.31	4.43 E-04
TIMP 4	12.51	3.30 E-04

Proteins and clinical covariates associated with post-operative hypoxemia burden (top) and length of stay (bottom) in the final selected multi-protein model. Considered clinical covariates included demographic and diagnostic variables. Positive coefficients indicate a direct relationship between the candidate predictor and outcome of interest while negative coefficients indicate an inverse relationship. MMP = matrix metalloprotease, FGF = fibroblast growth factor, 48h low sat% = percent of the first 48 post-operative hours with oxygen saturation < 70%. Adjusted R^2^ value reflects results of the final selected model including all variables listed.

**Figure 4:**
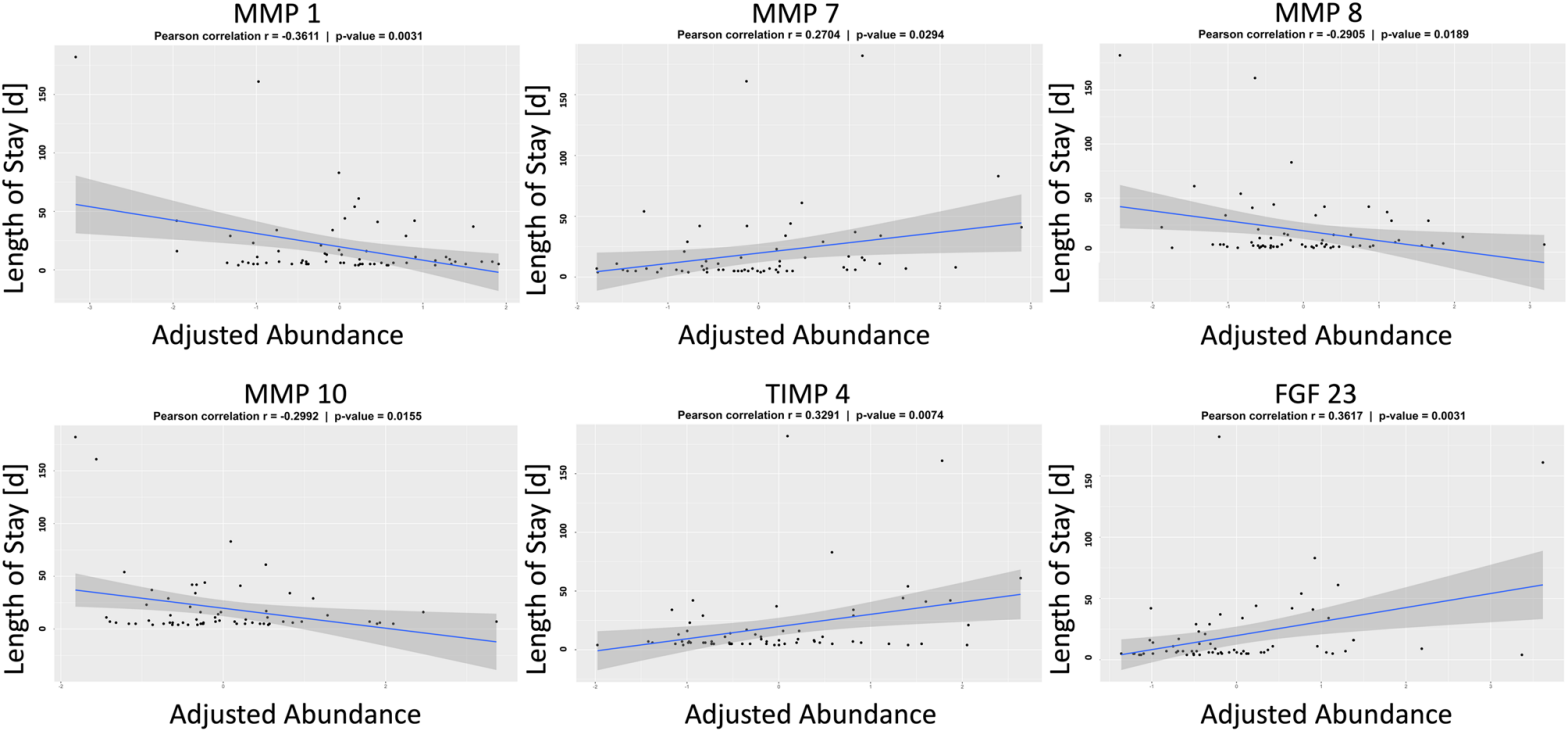
Association between proteins and length of stay. Scatterplots depict the log transformed, center scaled, heparin-status adjusted protein abundance and post-Stage 2 length of stay for individual study subjects. Best fit line and 95% confidence interval are overlaid to depict the relationship between the variables. MMP = matrix metalloprotease, FGF = fibroblast growth factor, TIMP = tissue inhibitor of metalloprotease.

We then turned our attention to post-operation LOS. After controlling for demographic covariates, testing the protein abundances one by one there were six that showed a significant linear association with LOS: MMP 1, MMP 7, MMP 8, MMP 10, TIMP 4, and FGF 23. Scatterplots depicting the relationship between each adjusted circulating protein abundance and LOS for the individual patients are shown in Fig. 4. The final selected multiple-protein model demonstrated a moderate ability to predict the outcome variable (R^2^ = 0.403, adjusted R^2^ = 0.363) and included four protein abundances (MMP 1, MMP 7, MMP 8, and TIMP 4; Table 3). MMP 7 and TIMP 4 levels were higher in subjects with longer LOS, while MMP 1 and MMP 8 levels were lower. Repeating this testing with additional consideration of the diagnostic variables as covariates the final winner multiple-protein model demonstrated a positive association between MMP 7 and TIMP 4 and LOS while MMP 1, MMP 8, and MMP 10 had a negative association with the outcome (full model R^2^ = 0.444, adjusted R^2^ = 0.394).

Finally, we evaluated whether circulating levels of any of the proteins of interest were associated with the secondary outcomes. Considering only the demographic variables as covariates, in the protein-by-protein analysis MMP 7 had a positive association and MMP 14 had a negative association with likelihood of discharge on a PDE-5i (*p* = 0.03 for both). On multiple-protein analysis considering the demographic and diagnostic variables, the final winner model included a positive association between MMP 7 and PVRi and discharge on PDE-5i, and a negative association between MMP 14 and PDE-5i use. Considering only the demographic variables in protein-by-protein analysis, we found a positive association between both MMP 7 (*p* = 0.01) and FGF 20 (*p* = 0.04) and total pleural drainage.

## Discussion

We present a prospective, cohort study of comprehensive mapping of circulating MMP, TIMP, and FGF protein family members in interstage infants with SVHD and healthy controls. We report (1) significant differences between cases and controls among multiple members of all 3 protein families including 20 of 39 targeted proteins, (2) a significant association between increased interstage levels of MMP 7, MMP 17, and FGF 22, and decreased levels of MMP 8, MMP 14, and FGFR2 and post-Stage 2 hypoxemia burden after controlling for clinical covariates, and (3) a significant association between increased interstage levels of MMP 7, TIMP 4, and FGF 23, and decreased levels of MMP 1, MMP 8, and MMP 10 and post-Stage 2 length of stay after controlling for clinical covariates. Multivariable modeling demonstrated that, after accounting for clinical covariates, MMP 7, MMP 8, MMP 17, and FGFR2 had a persistent relationship with hypoxemia while MMP 1, MMP 7, MMP 8, and TIMP 4 had a persistent relationship with LOS. These findings indicate an association between extracellular matrix dysregulation and adverse post-Stage 2 outcomes in SVHD.

### MMP, TIMP, and FGF dysregulation in interstage SVHD

In our cohort we noted significant changes in 50% of the measured MMP and TIMP family members in SVHD cases compared to healthy controls; for 19 of the 20 altered proteins, we found higher levels in the SVHD cohort than in the healthy children. The MMP family of proteins and their endogenous inhibitors, the TIMPs, are the major modulators of extracellular matrix composition in a variety of tissues^9^. Increased MMP activity has been demonstrated to be pathologic in the pulmonary vasculature of both animal and human subjects with pulmonary hypertension (PH)^6,18,19^. MMP 2 in particular is known to promote intimal hyperplasia and vascular remodeling in PH patients^6^. TIMP 1 levels are higher among adults with PH compared to healthy controls and TIMP 2 has shown an association with pulmonary arterial stiffness in children with PH^20,21^. Increased levels of multiple MMPs and TIMPs in our cohort (including MMP 2, TIMP 1, and TIMP 2) may, therefore, indicate increased extracellular matrix turnover, a pathologic change that could put interstage SVHD patients at risk for adverse pulmonary vascular outcomes.

The circulating concentration of 11 of the 21 tested circulating FGFs differed significantly between cases and controls, with higher levels seen among cases for all proteins. The FGFs are a functionally diverse group of 18 circulating growth factors, grouped into 6 subfamilies based on sequence homology and function, with important effects mediated through a variety of tissue-specific FGF receptors (FGFRs) ranging from angiogenesis to neurodevelopment and maintenance of homeostasis^11^. The 3 FGFs that were most significantly upregulated in SVHD cases compared to controls were FGF 4, 20, and 23. FGF 4 is a pro-angiogenic factor that has been studied as potentially protective in adults with insufficient coronary blood flow^11^. Higher levels among SVHD patients, therefore, may represent an adaptive response to improve sub-optimal tissue-level perfusion secondary to interstage physiology. FGF 20 has been linked to anti-inflammatory properties in intestinal tissues; its roles in the heart and lung are less well defined^22^. Finally, high levels of circulating FGF 23 are known to contribute to airway inflammation, endothelial dysfunction, endogenous nitric oxide dysregulation, myocardial hypertrophy, and cognitive dysfunction^23–26^. In our prior work, we demonstrated that post-Stage 2 circulating FGF 23 is one of the major drivers of the hypoxemic fingerprint^5^. On single biomarker testing, controlling for clinical covariates, in this study we found both elevated interstage FGF 23 in SVHD compared to controls and a linear association between pre-Stage 2 FGF 23 level and longer post-Stage 2 LOS within the SVHD infants. Put together, these findings suggest that elevated FGF 23 in SVHD both pre- and post-Stage 2 could be maladaptive and place patients at risk for intolerance of Stage 2 physiology. Future, mechanistic studies evaluating the cellular and tissue level effects of FGF dysregulation in SVHD will be important.

### Association between biomarkers and adverse outcomes

On single variable testing we found significant associations between pre-Stage 2 levels of multiple biomarkers of interest and each of our primary outcomes. Two proteins in particular were associated with both adverse outcomes of interest: increased MMP 7 and decreased MMP 8. Mechanistically, increased MMP 7 activity is thought to worsen both parenchymal lung disease and pulmonary vascular disease through promotion of endothelial to mesenchymal transition, increased fibrosis, and increased vascular smooth muscle cell proliferation^7,27^. Our prior work additionally demonstrated an association between increased post-operative MMP 7 and post-Stage 2 hypoxemia^5^. Increased circulating MMP 7 has also been associated with pulmonary pathology in a number of clinical populations, including idiopathic pulmonary fibrosis, chronic obstructive pulmonary disease, and pulmonary arterial hypertension^7,28–30^. In this context, our results suggest a role for increased MMP 7 exposure as both a biomarker of disease and a potential mechanistic driver of pulmonary vascular inadequacy in SVHD.

Circulating levels of MMP 8, while elevated in SVHD compared to controls, showed an inverse association with both length of stay and post-operative hypoxemia burden within the SVHD cohort. The association between higher MMP 8 levels and more favorable outcomes is aligned with prior mechanistic work suggesting a role for MMP 8 as pro-angiogenic and supportive of endothelial cell function^9^. Further, a recent translational article demonstrated that MMP 8 may be protective, opposing progression of pulmonary vascular disease in adult pulmonary hypertension patients^31^. The combination of elevated MMP 8 in SVHD compared to controls but an association between higher MMP 8 and more favorable outcomes suggests that failure to activate MMP 8 in a subset of the SVHD cohort may be a mechanistic driver of pulmonary vascular inadequacy. Collectively, our findings raise the possibility that an imbalance between the proliferative effects of MMP 7 and the antiproliferative effects of MMP 8 may be deleterious in interstage SVHD patients and is an important area for future mechanistic study.

Two additional biomarkers of interest, TIMP 4 and MMP 1, were directly associated with post-Stage 2 LOS. While the role of MMP 1 in the pulmonary vasculature is less clear, elevated TIMP 4 level has been previously identified as a biomarker of disease and indicator of poor prognosis in pulmonary arterial hypertension^30,32–34^. Although not evaluated previously in SVHD, a pediatric study linked elevated TIMP 4 levels to increased pulmonary vascular stiffness by MRI providing a potential mechanism linking TIMP 4 activity to impaired tolerance of Stage 2 physiology^20^.

We did not find evidence for a significant association between age at Stage 2, sex, or ventricular morphology and either outcome, a difference from some prior studies that have suggested prolonged LOS in younger patients undergoing Stage 2^35,36^. We did note an unexpected association between higher growth index (weight per height) and post-operative hypoxemia. As prior studies have shown smaller weight rather than larger weight to be a risk factor for adverse outcomes, this association may be driven by linear growth faltering in a subset of the SVHD cohort^37,38^. Further studies specifically targeting detailed assessment of growth parameters in interstage SVHD will be important.

### Limitations

This study has certain important limitations. All patients were cared for in Denver, approximately 1 mile above sea level, potentially affecting the ability to generalize our findings to patients cared for at lower elevation. This is a single center study in a rare disease, so the sample size is modest. Unmeasured variations in clinical practice and anatomy among patients in the cohort may confound our results, in particular the clinical outcomes of interest. We found evidence of lower levels for 3 proteins among subjects with pre-op samples collected during cath rather than in the OR. Evaluating whether precise timing of sampling or unmeasured clinical factors affecting availability of a cath sample may be confounding this association is an important area of future study. Studies using absolute quantification-based methodologies will be important to confirm our findings and establish optimal cutoff values to identify post-operative risk for the biomarkers of interest. The adjusted R^2^ values from the multivariable models are moderate suggesting that an important portion of the variability in the outcome variables discussed is derived from factors not assessed in this study. As our outcomes are entirely focused on post-Stage 2 recovery, longitudinal follow-up to evaluate how biomarker levels change over time is needed. Despite similar inclusion criteria the control subjects were slightly older than the SVHD cases, introducing age as a potential confounder in the case–control analysis.

### Summary

We report the novel finding that targeted, family-level mapping identifies broad alterations in circulating MMPs, TIMPs, and FGFs in interstage infants with SVHD compared to healthy controls. After adjusting for clinical covariates, single biomarker testing identified several targets significantly associated with either post-Stage 2 hypoxemia or LOS including FGF 22, FGF 23, FGFR2, MMP 1, MMP 7, MMP 8, MMP 10, MMP 14, MMP 17, and TIMP 4. Multivariable models accounting for key clinical and demographic variables identified increased circulating MMP 7 and decreased circulating MMP 8 as significantly associated with both hypoxemia and LOS. These findings suggest that dysregulation of extracellular matrix production, and specifically imbalance of MMP 7 and MMP 8, may be an important driver of post-Stage 2 morbidity in SVHD.

## Data Availability

The datasets analyzed during the current study are available from the corresponding author on reasonable request.

## Abbreviations

SVHD: Single ventricle heart disease
MMP: Matrix metalloproteinase
TIMP: Tissue inhibitor of metalloproteinases
FGF: Fibroblast growth factor
ECM: Extracellular matrix
CHD: Congenital heart disease
ETT: Endotracheal intubation time
BIC: Bayesian information criterion
48h low sat%: Percent of the first 48 post-operative hours with oxygen saturation < 70%
LOS: Length of stay
